# Supplementary figures and images for: A Drosophila model of Pontocerebellar Hypoplasia reveals a critical role for the RNA exosome in neurons
Source: PLoS Genet. 2020 Jul 9;16(7):e1008901. doi: 10.1371/journal.pgen.1008901 (PMC7373318; doi:10.1371/journal.pgen.1008901)

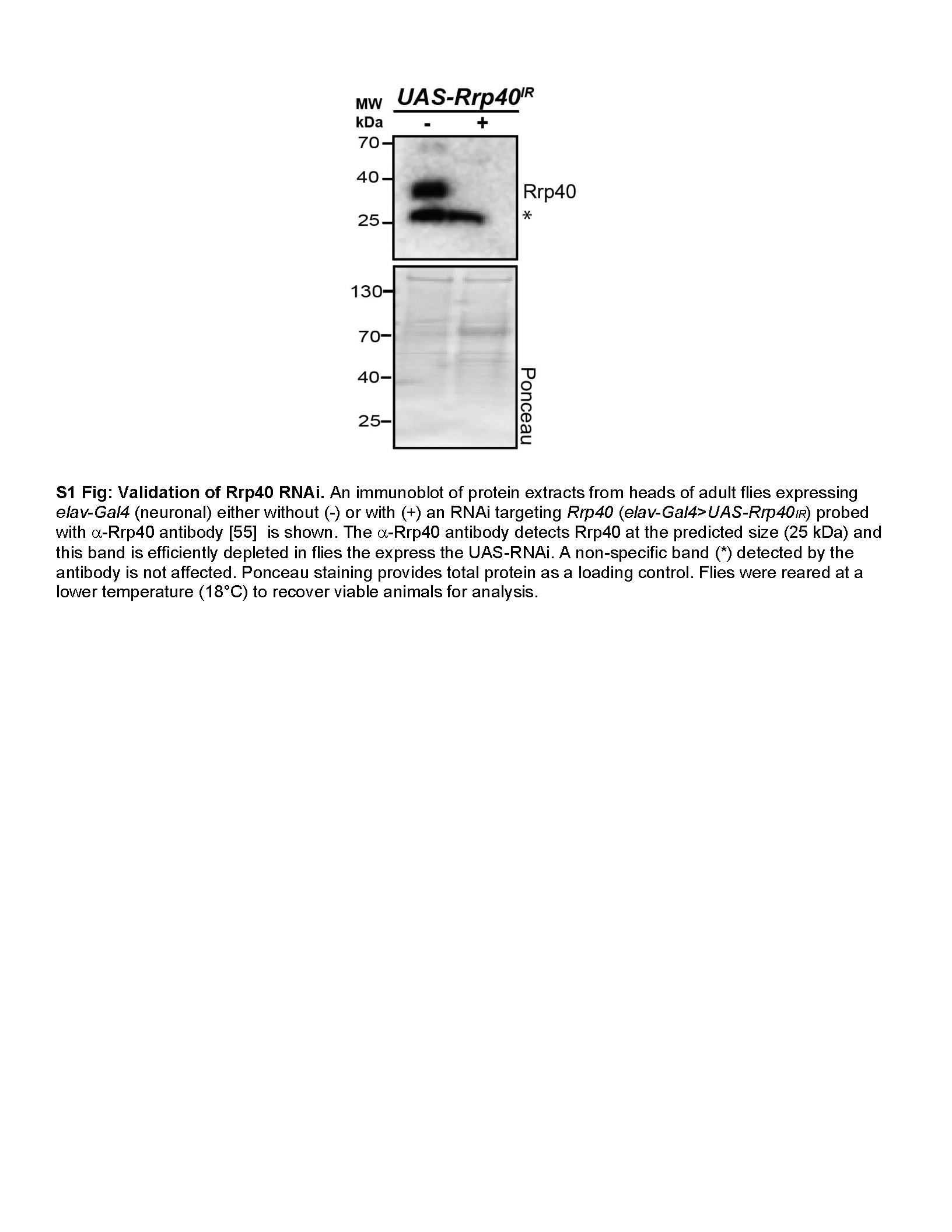

Supplement: S1 Fig — An immunoblot of protein extracts from heads of adult flies expressing elav-Gal4 (neuronal) either without (-) or with (+) an RNAi targeting Rrp40 (elav-Gal4>UAS-Rrp40IR) probed with α-Rrp40 antibody [55] is shown. The α-Rrp40 antibody detects Rrp40 at the predicted size (25 kDa) and this band is efficiently depleted in flies the express the UAS-RNAi. A non-specific band (*) detected by the antibody is not affected. Ponceau staining provides total protein as a loading control. Flies were reared at a lower temperature (18°C) to recover viable animals for analysis. (TIFF) [file pgen.1008901.s001.tiff]

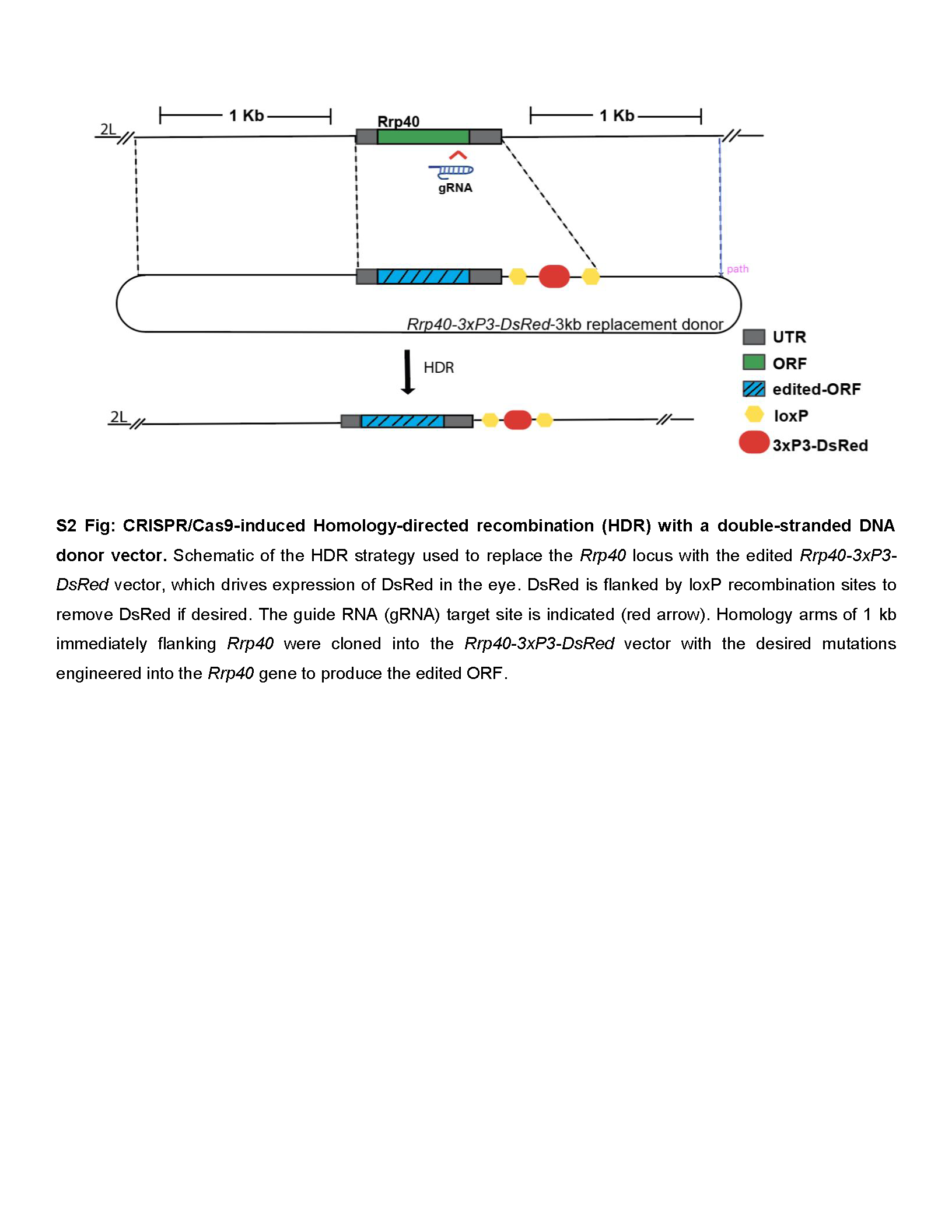

Supplement: S2 Fig — (A) Schematic of the HDR strategy used to replace the Rrp40 locus with the edited Rrp40-3xP3-DsRed vector, which drives expression of DsRed in the eye. DsRed is flanked by loxP recombination sites to remove DsRed if desired. The guide RNA (gRNA) target site is indicated (red arrow). Homology arms of 1 kb immediately flanking Rrp40 were cloned into the Rrp40-3xP3-DsRed vector with the desired mutations engineered into the Rrp40 gene to produce the edited ORF. (TIFF) [file pgen.1008901.s002.tiff]

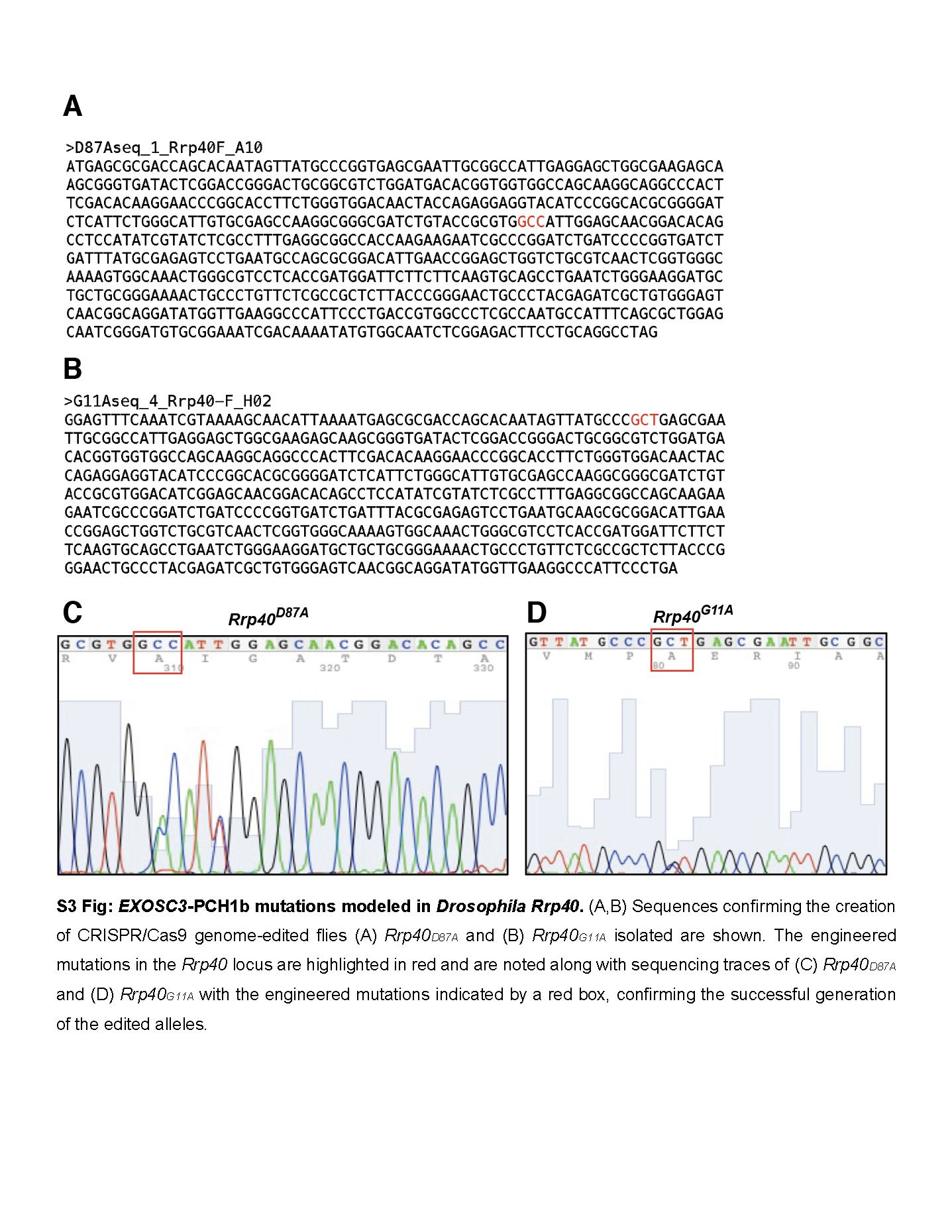

Supplement: S3 Fig — (A,B) Sequences confirming the creation of CRISPR/Cas9 genome-edited flies (A) Rrp40D87A and (B) Rrp40G11A isolated are shown. The engineered mutations in the Rrp40 locus are highlighted in red and are noted along with sequencing traces of (C) Rrp40D87A and (D) Rrp40G11A with the engineered mutations indicated by a red box, confirming the successful generation of the edited alleles. (TIFF) [file pgen.1008901.s003.tiff]

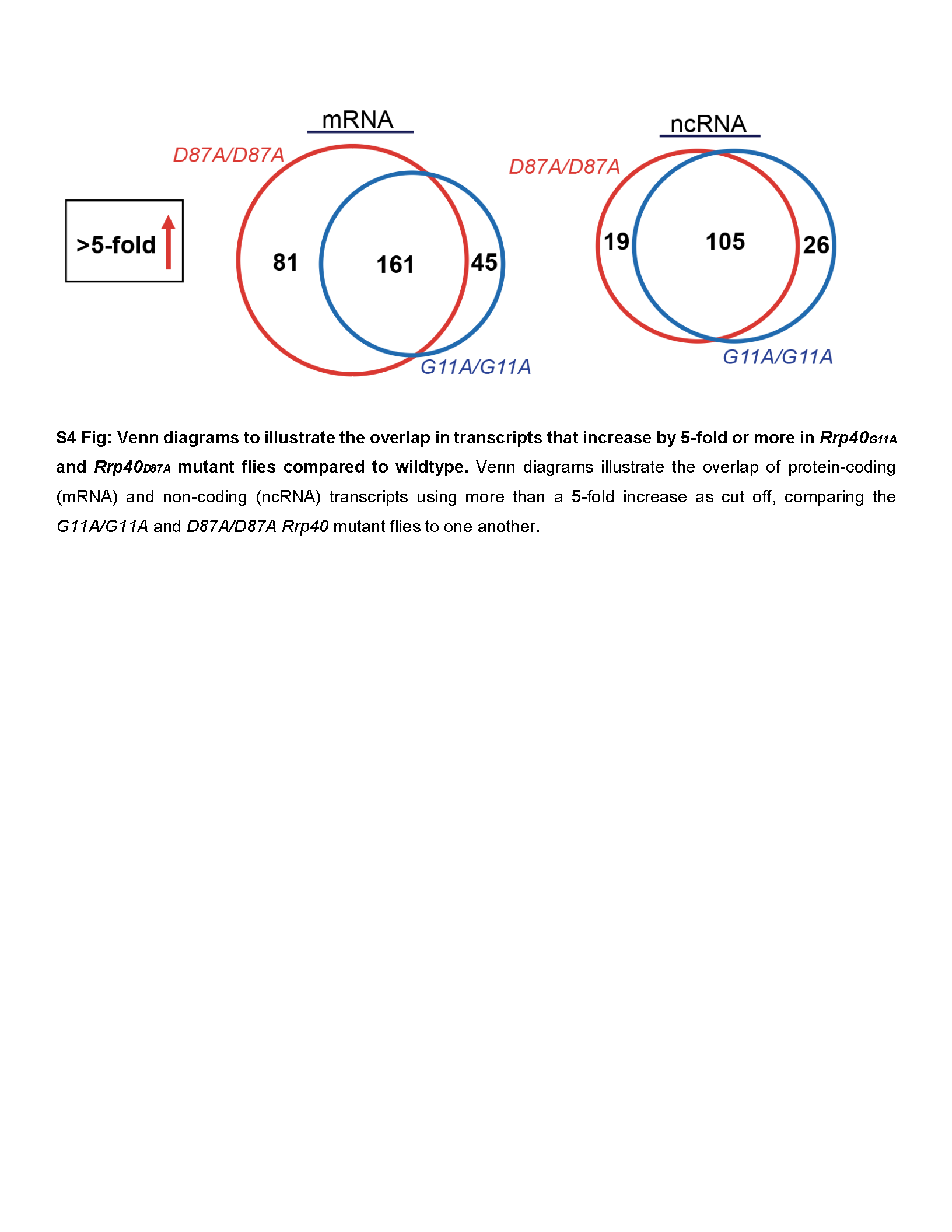

Supplement: S4 Fig — Venn diagrams illustrate the overlap of protein-coding (mRNA) and non-coding (ncRNA) transcripts using more than a 5-fold increase as cut off, comparing the G11A/G11A and D87A/D87A Rrp40 mutant flies to one another. (TIFF) [file pgen.1008901.s004.tiff]

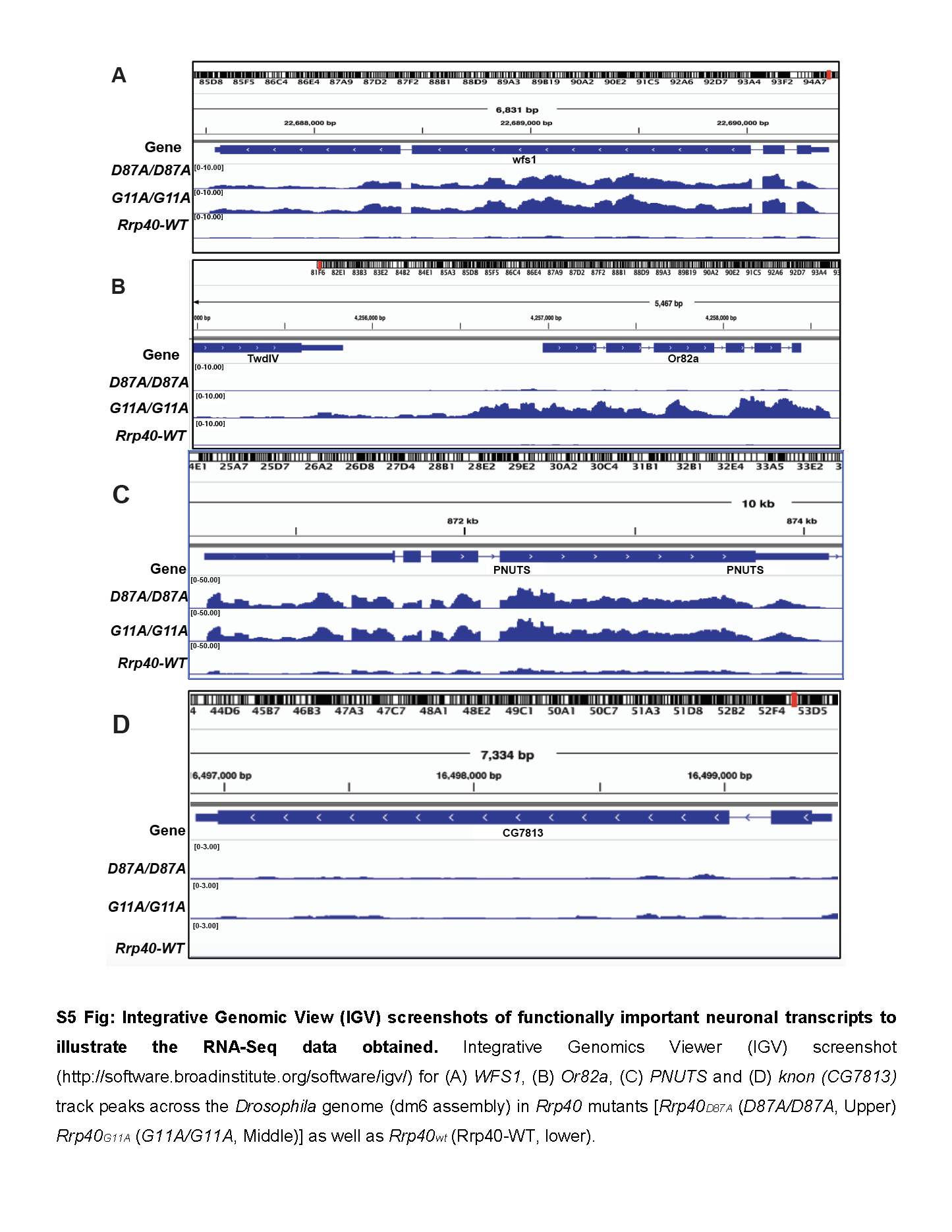

Supplement: S5 Fig — Integrative Genomics Viewer (IGV) screenshot (http://software.broadinstitute.org/software/igv/) for (A) WFS1, (B) Or82a, (C) PNUTS and (D) knon (CG7813) track peaks across the Drosophila genome (dm6 assembly) in Rrp40 mutants [Rrp40D87A (D87A/D87A, Upper) Rrp40G11A (G11A/G11A, Middle)] as well as Rrp40wt (Rrp40-WT, lower). (TIFF) [file pgen.1008901.s005.tiff]

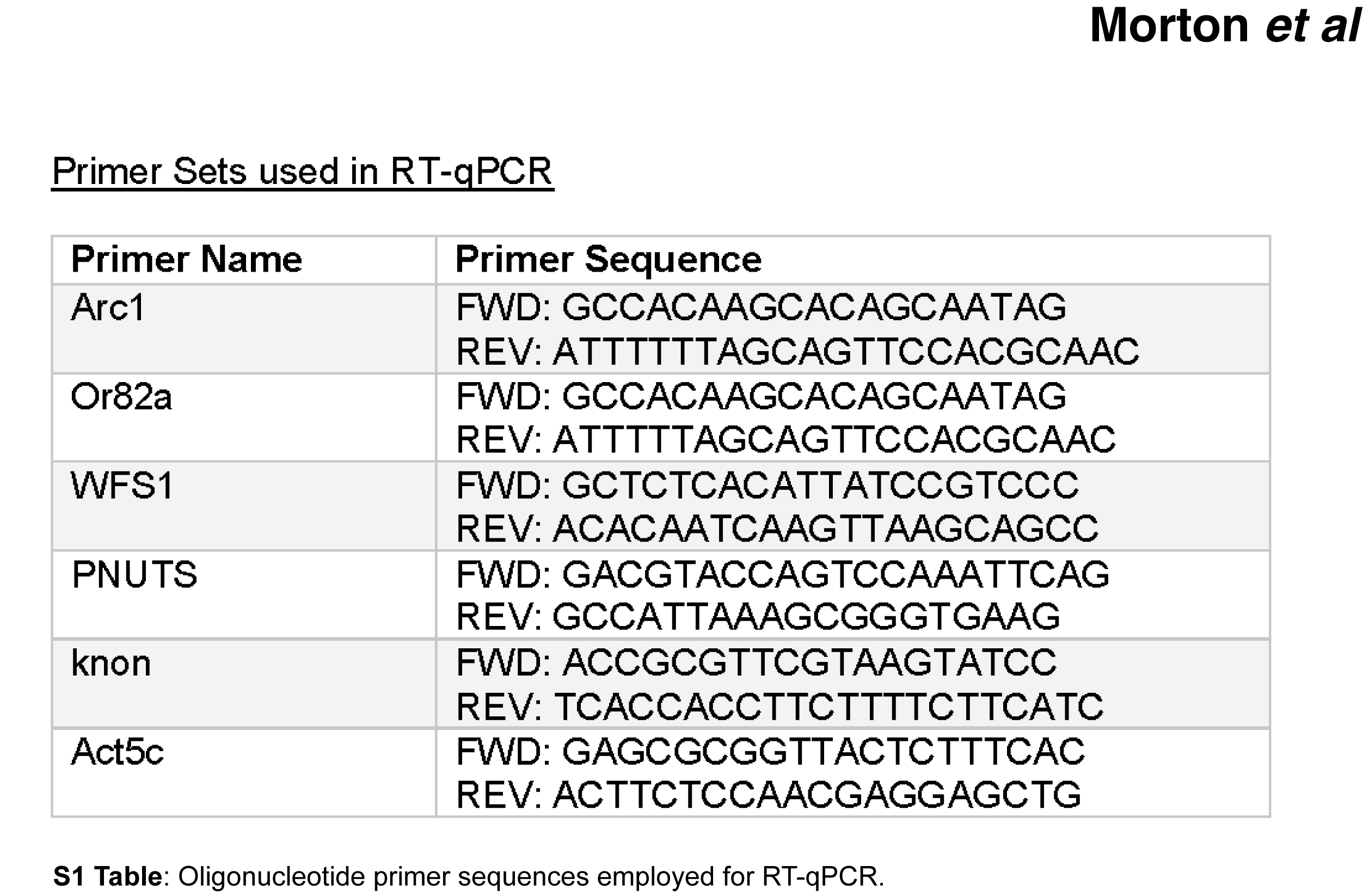

Supplement: S1 Table — (TIF) [file pgen.1008901.s006.tif]
