# Supplementary material for: A Drosophila model of Pontocerebellar Hypoplasia reveals a critical role for the RNA exosome in neurons
Source: PLoS Genet. 2020 Jul 9;16(7):e1008901. doi: 10.1371/journal.pgen.1008901 (PMC7373318; doi:10.1371/journal.pgen.1008901)
Supplement: Attachment — Submitted filename: FINAL Detailed Response to Reviewer Comments.docx [file pgen.1008901.s012.docx]

**Detailed Response to Reviewer Comments**

Reviewer #1: Morton et al. have investigated the neuronal requirements of EXOSC3 and the RNA exosome in a “Drosophila model of pontocerebellar hypoplasia (PCH1b)”. Recent evidence strongly implicates RNA metabolism in the pathogenesis of neurologic disorders and for neuronal maintenance. Given the expected broad requirement for exosome functions across all cell types, how genetic variants in this complex specifically affect neurons is an interesting unanswered question. Using a CRISPR strategy, Morton et al. introduce 2 PCH1b mutations into fly Rrp40, the ortholog of EXOSC3, and characterized resulting phenotypes affecting survival and locomotor impairment. Perhaps the most interesting and important finding is evidence to support an “allelic series” of phenotypic severity which shows some consistency with genotype-phenotype studies of human PCH1b patients. RNA-sequencing in the fly mutants also identify many transcripts showing increased abundance, consistent with altered stability/turnover, including in the synaptic regulator, Arc1.

Overall, this manuscript makes some important initial contributions to understanding the role of EXOSC3 in the adult brain and its potential impact on the transcriptome; however, it also leaves many questions unanswered, with 2 particular issues looming large. First, while the potential cross-species genotype-phenotype correlations are intriguing, the potential mechanism(s) of the PCH1b-causing variants remains unresolved. While the RNA-seq analyses confirm a global transcriptome perturbation, the allele with more severe phenotypes (G11A) has a less substantial impact on the transcriptome overall [number of transcripts affected and magnitude of change, Fig 6B vs. C]—this should be discussed. Notably, Arc1 does appear to be more severely affected in G11A, but this is correlative in the absence of additional evidence that Arc1 modifies Rrp40-dependent phenotypes. Since Rrp40 protein expression and that of several other exosome complex proteins appears unaffected (but see below), other in vivo or in vitro studies might be considered to provide clues as to the molecular mechanisms for these variants and the relative phenotypic strengths. Second, and perhaps even more importantly, since the manuscript purports to develop “A Drosophila model of pontocerebellar hypoplasia”, it is essential to determine if the model recapitulates the developmental and/or pathologic degenerative features that define this disorder. The phenotypes characterized here (survival, locomotor impairment, and wing posture) are rather non-specific. While the rescue studies support a neuronal origin, some further experiments might easily address whether the fly brain and motor neurons are normally developed and whether there is evidence of neurodegeneration with aging (e.g. adult brain histology in newly-eclosed and aged adults).

We thank the reviewer for his/her support of the manuscript— 1) Finding that the manuscript makes important initial contributions to understanding the role of EXOSC3 in adult brains and its potential impact on the transcriptome; 2) Pointing out that our model supports a genotype-phenotype correlation consistent with studies of human PCH1b patients; and detailed suggestions for further improvement. We have addressed all of the comments, many by including additional data. While the original manuscript submitted had 7 figures, the revised manuscript contains 9 figures (many with additional data included) and a mechanistic model (Figure 10).

**Major Concerns**

1. First, while the potential cross-species genotype-phenotype correlations are intriguing, the potential mechanism(s) of the PCH1b-causing variants remains unresolved.

This is a valid statement, but is also largely true for all other published alleles of genes encoding RNA exosome subunits that have been linked to disease [1-4]. To address the question of mechanism, we provide a model as the final figure (Fig 10), and discuss possible pathogenic mechanisms in the Discussion. In addition, we make clear what aspects of this model we have addressed in the present study. While we have not identified a definitive mechanism underlying pathology, we provide evidence to eliminate some potential models. For example, a simple model such as a decrease in the level of the subunit or complex is unlikely to be sufficient to explain the phenotypes observed because, while we do detect a modest decrease in Rrp40 levels in the PCH1b model flies (~30%), we detect no obvious phenotypes in flies heterozygous for a deficiency that contains *Rrp40* (a 50% decrease). Defining how the specific amino acids alter the function of EXOSC3 is an important goal for the future. Genetic screens in *Drosophila* that exploit the *Rrp40* mutants described here could be an excellent way to approach this mechanistic question. Indeed, these mutants represent the first alleles produced of any structural subunit of the RNA exosome in flies, opening the door to understanding important fundamental aspects of RNA exosome function.

2. While the RNA-seq analyses confirm a global transcriptome perturbation, the allele with more severe phenotypes (G11A) has a less substantial impact on the transcriptome overall [number of transcripts affected and magnitude of change, Fig 6B vs. C]—this should be discussed. Notably, Arc1 does appear to be more severely affected in G11A, but this is correlative in the absence of additional evidence that Arc1 modifies Rrp40-dependent phenotypes.

We have analyzed the differences in transcript changes between *G11A* and *D87A* flies. For those transcripts that show a 5-fold or more increase in steady state levels, there is indeed no statistically significant difference in the number of transcripts changed in the two mutants (data presented in Fig 8B and 8C in the revised manuscript). However, the magnitude of these effects differs substantially between *G11A* and *D87A*. An excellent example of this is *Arc1* mRNA, which is increased 30-fold in *G11A* flies vs. 16-fold in *D87A* flies relative to controls. Thus, we speculate that differences in the magnitude of effect of many elevated RNAs, like *Arc1*, underlie differences in phenotype severity between *G11A* and *D87A*. We have tested whether decreasing the dose of *Arc1* with the *Arc1^[esm18]^* allele rescues *G11A* phenotypes, but only observed a modest effect (data not shown). As *Arc1* l is one of 336 transcripts that shows a greater than 5-fold change in *G11A* flies, we infer that the *G11A* phenotype is the cumulative effect of changing the level of many individual RNAs (e.g. see Fig 10) and that reducing one target is insufficient to rescue *G11A* animals. Future studies could test more comprehensively whether any particular candidate target transcripts play disproportionate roles in the *G11A* and *D87A* phenotypes.

3. Since Rrp40 protein expression and that of several other exosome complex proteins appears unaffected (but see below), other in vivo or in vitro studies might be considered to provide clues as to the molecular mechanisms for these variants and the relative phenotypic strengths.

We definitely agree that this is a goal for future studies and we are using a variety of model systems to approach this mechanistic question [5]. With this study, we are in a position to add genetic screens in *Drosophila* to the toolbox for future studies. Indeed, the mechanistic model presented as Figure 10 and accompanying discussion address this goal.

- Also alluded to above, western blot evidence is provided (Fig 5) suggesting that the Rrp40 alleles created do not significantly affect protein expression; however, the data does not allow assessment of a more modest, but significant impact. This is an important experiment, and it would be advisable to perform several replicates and provide a quantification with statistical analysis.

At the reviewer’s suggestion, we have now repeated and quantified the immunoblots analyzing PCH1b mutation effects on RNA exosome subunit protein levels. These results are presented as Figure 7B in the revised manuscript. These experiments are challenging because they rely on antibodies generated some time ago [6] which were generously provided to us after a deep freezer dive. While we do detect a statistically significant decrease in levels of both Rrp40 and Rrp4 (another cap subunit), the decrease detected is comparable in the two *Rrp40* mutants analyzed (no statistically significant difference), which suggests this decrease does not drive phenotypic differences. Furthermore, flies heterozygous for a deficiency (*Df(2L*), which removes a region of the genome that encodes Rrp40 show no obvious phenotypes. These flies should have a 50% decrease in the dose of Rrp40, which is less than the ~30% decrease measured in the CRISPR alleles, suggesting that a modest decrease in Rrp40 is not sufficient to drive phenotypes. Instead, the specific amino acid changes are likely to cause functional consequences that differ due to the specific positions and nature of those changes.

- Also, have prior studies from human tissue or cell culture shown consistent results?—if yes this should be mentioned. This result might be considered to present earlier (with Fig 2).

To address the reviewers question regarding prior studies from human tissue or cell culture analyzing effects of PCH1b variants on protein stability- A previous study using patient fibroblast or myoblasts to examine variants of EXOSC8 (a core ‘ring’ subunit) linked to another form of PCH, revealed not only reduced EXOSC8 variants, but also EXOSC3 (Rrp40), indicating that defects in core ‘ring’ subunits of the RNA exosome can lead to a reduction in other subunits [1,2]. This finding is rather surprising given that the patients analyzed did not exhibit pathology related to the cell types analyzed. Our findings suggest that a simple loss of the altered subunit is not sufficient to explain phenotypes observed at least in the *Drosophila* model. Such models also do not explain why mutations in different genes that encode structural subunits of the RNA exosome cause distinct clinical presentations.

- If the Rrp40 variants do not affect protein expression or stability, it would be informative to address other possible mechanisms. Are immunoprecipitation experiments feasible to address whether the complex is intact? Other possibilities are to examine localization (in vivo) or RNA-binding or other assay for activity (in vitro). At a minimum, the possible mechanisms should be thoroughly discussed, including future experimental directions that would be helpful to resolve.

The reviewer makes an excellent point that additional biochemical analysis may reveal whether or not PCH1b mutations affect overall RNA exosome complex integrity. However, our current model system is not ideal for the biochemical experiments necessary to answer these questions. Furthermore, we are limited by the lack of reagents (such as antibodies) needed to carry out these experiments. To address the reviewer’s recommendation, we have added a mechanistic model (Fig 10) and modified the Discussion to address possible mechanisms by which PCH1b missense mutations could affect RNA exosome function. We have addressed aspects of this model in the current study because our data suggests that a simple decrease in the amount of the subunit does not explain phenotypic differences (Fig 7) and we have also provided the first ever list of potential RNA exosome targets from a neuronally-enriched tissue (Fig 8).

4. Second, and perhaps even more importantly, since the manuscript purports to develop “A Drosophila model of pontocerebellar hypoplasia”, it is essential to determine if the model recapitulates the developmental and/or pathologic degenerative features that define this disorder. The phenotypes characterized here (survival, locomotor impairment, and wing posture) are rather non-specific. While the rescue studies support a neuronal origin, some further experiments might easily address whether the fly brain and motor neurons are normally developed and whether there is evidence of neurodegeneration with aging (e.g. adult brain histology in newly-eclosed and aged adults).

- As noted above, studies should be performed to broadly survey structure and maintenance of adult brain structures to determine whether neurodevelopmental and/or degenerative aspects of PCH1b are recapitulated (e.g. studies in young and aged adults). It may also be informative to examine other accessible nervous system structures, including the larval neuromuscular junction, since spinal motor neuron degeneration is a defining feature of PCH1b (based on OMIM); quick surveys of the adult retina and larval brain may also be informative.

The reviewer makes an excellent point that additional experiments are needed to address whether our PCH1b model flies have normally developed brains or neurodegeneration with aging. We have addressed this question here by performing whole brain dissections, immunostaining, and confocal microscopy to explore the requirement for Rrp40 in the brain with several different conditions to deplete Rrp40 (New Figures 1C and 2D) or analyze the alleles we have produced (New Figure- Figure 5).

First, we exploited a structure in the fly brain that is not essential for viability, the Mushroom Body (MB), and studied whether depletion of Rrp40 affects MB morphology (Fig 1C). This analysis reveals defects in the MB morphology upon depletion of Rrp40.

We next employed a temperature sensitive RNAi driver to obtain flies with Rrp40 depleted from all neurons. In the small cohort of flies that eclose, we detect significant morphological defects in MBs (Fig 2D).

In addition to our studies analyzing the effects of Rrp40 depletion in different sets of neurons, we also analyzed the brains of the *Rrp40* mutant flies we generated (Fig 5). This analysis revealed that flies with the most severe missense mutation (*Rrp40^G11A^*) show defects in MB morphology involving β-lobe neurons (Fig 5).

5. The RNA-interference experiments appear to based on a single transgenic line, raising the possibility of off-target effects. Did the authors validate by western blot or RT-PCR that RNAi reduces Rrp40 expression?—this would also enhance interpretation of Fig 5.

The reviewer is correct that the RNAi experiments are based on the single transgenic RNAi line available, which could potentially raise the possibility of off-target effects. Unfortunately, there is only one Rrp40 RNAi line commercially available, limiting our ability to definitively rule out off-target effects with this approach. To validate the RNAi line, we now provide experimental evidence for the successful knockdown of Rrp40 as confirmed by immunoblotting (S1 Fig). Importantly, our results with RNAi-mediated depletion of Rrp40 parallel previous studies that used various drivers to deplete other RNA exosome subunits [4,7], albeit these studies primarily focused on viability. Finally, given that the majority of the manuscript (7 of 9 data figures) is focused on analysis of CRISPR engineered *Rrp40* alleles and transgenic rescue of these alleles is performed, we would argue that most phenotypes characterized cannot result from off target effects of the RNAi.

6. The table in fig. 4A appears to largely present negative data—the absence of rescue for semi-lethality, but this was not addressed further in the text. Some context and interpretation should be provided. Do ubiquitous GAL4 drivers (e.g. act5c) rescue viability? In a related question, did the authors consider attempting rescue of the locomotor phenotype using a PNS, motor neuron, or muscle specific driver line? Motor neuron might be especially meaningful given the involvement in PCH1b.

We have now addressed the failure of transgenic EXOSC3 to rescue the semi-lethality that is presented in revised Fig 6A (was 4A) in the text by speculating about an early developmental requirement for Rrp40 that cannot be supplied by human EXOSC3. As suggested by the reviewer, we repeated rescue experiments using a muscle-specific driver, which did not rescue locomotor defects. These data are presented as part of Fig 6B in the revised manuscript.

7. How did the authors choose to focus on the specific transcripts presented in Fig. 7 and in the results text (first paragraph of “steady state levels of key neuronal genes” section)? Without a systematic description, it appears that this subgroup was “cherry-picked”. Importantly, are there specific sequence features that suggest RNAs affected by Rrp40 alleles are targeted by the exosome? It appears that exosome may target RNAs containing AU-rich elements in 3’UTRs—can this be experimentally validated from the RNAseq data. Given the involvement of the exosome in nonsense mediated decay, one might expect the accumulation of transcripts containing errors, or secondary changes in other proteins that respond to transcriptional errors.

While some recent studies of the RNA exosome linked to disease pathology have focused on AU-rich element-containing RNAs [2], the RNA exosome targets many RNAs with no established enrichment for AU-rich-containing transcripts. We did perform an analysis to identify any enrichment for AU-rich transcripts in our set of transcripts that show a statistically significant change, but there was no statistically significant enrichment for such transcripts. This finding is consistent with the fact that the RNA exosome primarily functions as a 3’-5’ exonuclease without binding site specificity. There are limited studies that define RNA exosome targets in *Drosophila* S2 cells [8] and no previous studies that employ any hypomorphic alleles of genes encoding structural subunits of the RNA exosome in any *in vivo* multicellular model (there are a number of elegant experiments in budding yeast). Here, we provide a list of all differentially expressed genes in the supplemental data and then focus on some that show the greatest changes such as *Arc1* and *Or82a*. Other transcripts were selected due to important neuronal functions.

8. Are there available data from human studies (either cells or postmortem tissue) that can inform on whether the RNA transcripts that change in Rrp40 mutants represent either conserved transcriptomic features of PCH1b or exosome failure. Are the genes disrupted in fly heads conserved and expressed in human neuronal tissues that are impacted in PCH1b? Is this first transcriptome-wide analysis of consequences following genetic disruption of the RNA exosome? If yes, this should be made clear, as it will increase interest. If not, than it is important to compare the transcripts affected with other available datasets.

The reviewer raises an excellent point in inquiring whether or not there are existing datasets (either cells or postmortem tissue) that can inform whether RNA transcripts that change in *Rrp40* mutants represent conserved features of PCH1b or RNA exosome defects- unfortunately there have not been any prior studies that have examined the transcriptome of PCH1b models. We have now clarified that this is the first transcriptome-wide analysis of consequences following genetic disruption of the RNA exosome (beyond RNAi-mediated depletion in various cell types). There is one study published since the initial submission of this manuscript that depleted another RNA exosome subunit, EXOSC2, in *Drosophila* and performed transcriptomic analysis [4]. We are attempting to obtain the raw data from these authors to compare to our results. This analysis would be particularly interesting as mutations in EXOSC2 cause a distinct clinical presentation with little relation to PCH1b [3,9] . Importantly, these authors did not model disease alleles as we have done here.

**Other issues for consideration:**

1. It would be helpful to include a bit more detail in the introduction describing the PCH1b disease phenotype, including developmental and degenerative aspects, and the specific observations that support a genotype-phenotype correlation—this is important to interpret the value of fly model.

We have provided some additional detail describing PCH1b disease phenotypes to underscore the importance/relevance of the fly model. We have expanded upon the description of these phenotypes in the introduction. In addition, we refer the reader to several recent reviews that address clinical presentation for this disease [9,10].

2. Please add to background or results the overall similarity and identity by amino acid sequence of Rrp40 in flies with human EXOC3.

We have updated the sequence alignment in Fig 3A (in the revised manuscript) to include the overall amino acid sequence identity/similarity of Rrp40 in flies and human EXOSC3.

3. Was any analysis of heterozygous animals performed (G11A/+ or D87A/+) to exclude potential dominant negative effects?

Yes, experiments were performed to ensure there are no dominant negative effects heterozygous flies (G11A/+ or D87A/+). This point is now mentioned in the Results section when the models are introduced.

4. The significance of the “wing posture” phenotype is obscure. Can the authors provide citations to support the statement that this is “consistent with neurological deficits”?

The section referring to both wing posture phenotypes and locomotor phenotypes being consistent with neurological deficits has been updated in the text with the appropriate references [11,12].

5. “Seizure-like behavior” is not immediately obvious in the provided video S1; It would be preferable to describe the behavior rather than use the descriptor “seizure-like”, which may not mean the same thing to all readers.

The term “Seizure-like behavior” has been removed and replaced with “…genotypes also display bouts of spasm interposed with periods of paralysis…”

6. Maybe mention the age of the adult flies used for RNA-seq studies in the results?

We have updated the RNA-seq studies results section to include the age of the flies. “To complement our functional studies, we performed RNA-Seq analysis on three biological replicates of adult heads for each of the following genotypes (*Rrp40^wt^*, *Rrp40^G11A^* and *Rrp40^D87A^*) from newly eclosed (Day 0) flies.” The experiments were performed with Day 0 flies because some of the genotypes have short lifespans that makes collecting the number of flies required at subsequent days challenging. Furthermore, our goal was to identify the most direct targets of the RNA exosome by analyzing transcriptome-wide differences at this time point before age-dependent phenotypes worsen and the potential for indirect changes increases.

7. The y-axis scale is highly variable in Fig 7 B-D. it would be more clear to show a common scale so the relative magnitude of RNA expression changes can be appreciated.

In addition to the main text figure with mixed scales, we added a supplemental figure (S6 Fig) with common scales for these data.

8. It will be helpful to provide full genotypes in figure legends or methods. The genotypes for controls in some studies are not clear (Fig 1E, 4B, 7D).

We have clearly defined all genotypes in the Figure Legends. For most studies with the engineered *Rrp40* mutant alleles, we have used a control that was edited in exactly the same manner as the PCH1b model alleles, with no mutation introduced (Rrp40-WT). We describe this control in the Results section and compare lifespan of this engineered wildtype control to *w^1118^* flies (Fig 3C).

9. There does not appear to be a section in methods or statements in figure legends adequately describing the statistical methods (e.g. Fig. 1E, 2B/C, 3B, and 4A). Were appropriate post-hoc tests used to adjust for multiple comparisons (e.g. Fig 7)?

We have updated the Materials and methods with a statistical analysis section describing the appropriate statistical tests. We have also included this information in the Figure Legends.

10. Please include scale bars for the IGV plots (Fig 7A, S5).

All IGV plots have been updated with the appropriate scale ranges.

11. Please provide column headers to aide interpretation of Table S4.

Table S4 has been removed.

Reviewer #2: Overview

In this manuscript, Morton et al characterize the in vivo function the Rpr40 gene in Drosophila, which encodes a conserved component of cap region of the RNA exosome complex. To study the roles of Rpr40, they first conduct in vivo RNAi experiments across different tissues, using the Gal4-UAS system, revealing a particularly strong deleterious effect of Rpr40 loss-of-function (LOF) in neuronal and glial cells on post-embryonic viability. Since the human orthologue of Rpr40, EXOSC3, has been implicated in the neurological disorder Pontocerebellar Hypoplasia Type 1b (PCH1b), using CRISPR/Cas9 technology they also created Prp40 variant fly models bearing PCH1b-associated mutations. These mutant strains have reduced viability and lifespan, with survivors displaying wing abnormalities and behavioural defects consistent with neurological impairments. Finally, their comparative transcriptomic analyses of mutant and wildtype flies identified several hundred upregulated mRNAs associated with gene regulatory functions (e.g. DNA binding, helicase activity, RNA binding), as well as several neuronal transcripts (e.g. Arc1, Or82A). Altogether, this study offers a characterization of new Drosophila models of PCH1b, while also revealing the tissue-type specific requirements for RNA exosome components.

General Assessment
Overall, this paper presents a well-executed set of experiments that employ gold-standard approaches (e.g. CRISPR genome editing) to address the in vivo functions of Prp40 in Drosophila. The finding that Prp40 LOF has a more severe effect in neurons and glial cells is certainly interesting. The study could be strengthened by further evidence to indicate that the observed effect are not due to extra-exosomal functions and by providing evidence of the impact of Prp40 mutations on neuronal and glial cell phenotypes.

We thank the reviewer for his/her support of the manuscript stating that paper presents well-executed experiments that employ gold-standard approaches. We appreciate the reviewer’s suggested additional experiments/edits to strengthen the manuscript. We have addressed all of the comments, many by including additional data.

Specific Comments

1. The paper should include ‘cellular level’ analyses to look at the phenotypes of neuronal or glial cell populations across developmental times points, e.g. in embryo or larval specimens. Do specific neuronal or glial cell populations display morphological abnormalities that would be consistent with altered expression of some of the transcripts that are found to be upregulated by RNA-seq?

Although, developmental experiments would provide additional critical insight into RNA exosome requirements across different cell types, the goal of this study was to provide the first *in vivo* model to assess the consequences of mutations in genes that encode RNA exosome subunits. We agree with the reviewer that “cellular level” experiments would provide additional insights, but we have taken advantage of *in vivo* tools to begin to define cell-type specific requirements for Rrp40 function. Our studies examine roles in neurons, glia, and muscle. While the *in vivo* studies do not permit conclusions about whether functions are cell autonomous, they do provide a new window into the function of the RNA exosome in vivo and the functional consequences of missense mutations that occur in patients. Indeed, this study creates the first genetic loss of function mutations in any structural RNA exosome subunit gene in a multicellular organism *in vivo*.

2. Could the authors explain their interpretation of why glial cell depletion would severely impact fly eclosion, with little effect on lifespan?

The short answer is no, but we speculate that there is an early essential developmental requirement for the RNA exosome that is critical in many tissues, including neurons and glia. When that requirement is bypassed, we speculate that the role of the RNA exosome is more critical for homeostasis in neurons than in glia. This speculation is consistent with the fact that transgenic expression of human EXOSC3 in neurons cannot rescue eclosion defects, but can rescue locomotor defects (revised Figure 6A,B).

3. Is there a reason why G11A/Df(2L) flies were not included in the analysis presented in Fig.2C?

G11A/Df(2L) does not model a genotype that has been reported in patients with PCH1b, therefore we did not originally add/include this genotype in the figure presented in the main text. However, we have now updated the figure with the G11A/Df(2L) genotype for clarity/consistency (Fig 3B), indicating that such a genotype has not been detected (ND) in patients. Interestingly, consistent with the genotype/phenotype correlation, flies with this genotype are not viable so this is the most severe genotype analyzed in flies and also one for which no patients have been reported. We thank the reviewer for the suggestion to include these data as this adds a data point to the genotype/phenotype correlation.

4. One question that emerges when considering RNA exosome components is whether there is any evidence for extra-exosomal functions that could potentially explain some of the phenotypes observed in the study? The authors should comment on this and, if feasible, provide some data to address what proportion of Rpr40 (either wt or mutant forms) are associated with other components of the exosome (e.g. gel filtration chromatography, co-IPs…).

While there has been some speculation that there could be functions for RNA exosome subunits outside the complex [13], this is a controversial idea that is not supported by biochemical evidence. Several studies show that when individual subunits are not incorporated into the RNA exosome complex, they are rapidly degraded [2,5,14]. Of note, depletion of all RNA exosome subunits depleted thus far causes early lethality. This does not eliminate the possibility of extra-exosomal function, but supports the idea that all the subunits could function within a single complex. Regardless, our current model system is not ideal for the biochemical experiments necessary to answer these questions. Furthermore, we are limited by the lack of reagents needed to carry out these experiments. Thus, a future goal is to employ different models such as cultured cells to explore the biochemical consequence(s) of these amino acid substitutions. To address the reviewer’s point here, we have added a model as Figure 10 and discussed possible mechanisms by which PCH1b missense mutations could affect RNA exosome complex integrity.

5. There seem to be some inconsistencies with the numbers of transcripts listed between the various diagrams in Fig. 6, i.e. the number of upregulated transcripts don’t match between panels B-C and D-E. The authors should double check these numbers and clarify things in the results section if necessary.

We thank the reviewer for noting this discrepancy, which resulted from using slightly different sets of data. We have corrected the error and ensured consistency across all results reported.

Typos:

p.2, files should be flies

‘files’ has been corrected to ‘flies’

p.8, nulls alleles should be null alleles.

‘nulls alleles’ has been corrected to ‘null alleles’

Reference cited:

1. Burns D, Donkervoort D, Bharucha-Goebel D, Giunta M, Munro B, et al. (2017) A recessive mutation in EXOSC9 causes abnormal RNA metabolism resulting in a novel form of cerebellar hypoplasia/atrophy with early motor neuronopathy. Neuromuscul Disord 27: S38.

2. Boczonadi V, Muller JS, Pyle A, Munkley J, Dor T, et al. (2014) EXOSC8 mutations alter mRNA metabolism and cause hypomyelination with spinal muscular atrophy and cerebellar hypoplasia. Nat Commun 5: 4287.

3. Di Donato N, Neuhann T, Kahlert AK, Klink B, Hackmann K, et al. (2016) Mutations in EXOSC2 are associated with a novel syndrome characterised by retinitis pigmentosa, progressive hearing loss, premature ageing, short stature, mild intellectual disability and distinctive gestalt. J Med Genet 53: 419-425.

4. Yang X, Bayat V, DiDonato N, Zhao Y, Zarnegar B, et al. (2019) Genetic and genomic studies of pathogenic EXOSC2 mutations in the newly described disease SHRF implicate the autophagy pathway in disease pathogenesis. Hum Mol Genet.

5. Fasken MB, Losh JS, Leung SW, Brutus S, Avin B, et al. (2017) Insight into the RNA Exosome Complex Through Modeling Pontocerebellar Hypoplasia Type 1b Disease Mutations in Yeast. Genetics 205: 221-237.

6. Andrulis ED, Werner J, Nazarian A, Erdjument-Bromage H, Tempst P, et al. (2002) The RNA processing exosome is linked to elongating RNA polymerase II in Drosophila. Nature 420: 837-841.

7. Lim SJ, Boyle PJ, Chinen M, Dale RK, Lei EP (2013) Genome-wide localization of exosome components to active promoters and chromatin insulators in Drosophila. Nucleic Acids Res 41: 2963-2980.

8. Kiss DL, Andrulis ED (2010) Genome-wide analysis reveals distinct substrate specificities of Rrp6, Dis3, and core exosome subunits. RNA 16: 781-791.

9. Fasken MB, Morton DJ, Kuiper EG, Jones SK, Leung SW, et al. (2020) The RNA Exosome and Human Disease. Methods Mol Biol 2062: 3-33.

10. van Dijk T, Baas F, Barth PG, Poll-The BT (2018) What's new in pontocerebellar hypoplasia? An update on genes and subtypes. Orphanet J Rare Dis 13: 92.

11. Howse PE (1975) Brain structure and behavior in insects. Annu Rev Entomol 20: 359-379.

12. Zhang YQ, Bailey AM, Matthies HJ, Renden RB, Smith MA, et al. (2001) Drosophila fragile X-related gene regulates the MAP1B homolog Futsch to control synaptic structure and function. Cell 107: 591-603.

13. Graham AC, Kiss DL, Andrulis ED (2009) Core exosome-independent roles for Rrp6 in cell cycle progression. Mol Biol Cell 20: 2242-2253.

14. Estevez AM, Lehner B, Sanderson CM, Ruppert T, Clayton C (2003) The roles of intersubunit interactions in exosome stability. J Biol Chem 278: 34943-34951.
